# Seasonal Initiation of Hemodialysis: A Single-Center Experience

**DOI:** 10.3390/jcm15208139

**Published:** 2026-10-20

**Authors:** Derya Başak Tanburoğlu, Emrah Günay, Hatice Geçol, İbrahim Dayanan, Muhammed Emin Arslan, Gökçe Paşa

**Affiliations:** 1 Department of Nephrology, Adana City Training and Research Hospital, 01230 Adana, Türkiye; egnay01@gmail.com; 2 Department of Internal Medicine, Adana City Training and Research Hospital, 01230 Adana, Türkiye; gecolhatice@gmail.com (H.G.); ibrahimdayanan56@gmail.com (İ.D.); drmearslan@gmail.com (M.E.A.); 3 Department of Internal Medicine, Erzin State Hospital, 31960 Hatay, Türkiye; gokce.pasa@hotmail.com

**Keywords:** hemodialysis, seasonal variation, vascular access, catheter dysfunction, emergency dialysis initiation

## Abstract

**Objective**: This study aimed to evaluate the seasonal pattern of hemodialysis initiation in a warm/subtropical climate region (Adana) and its association with the mode of initiation, early complications, and mortality. **Materials and Methods**: A total of 319 patients who started chronic hemodialysis at Adana City Training and Research Hospital, Department of Nephrology, between 2022 and 2025 were retrospectively analyzed. Chi-square/Fisher’s exact and Kruskal–Wallis tests were used for seasonal comparisons. Multivariable logistic regression models adjusted for age, sex, diabetes, hypertension, and cardiovascular disease (coronary artery and/or valvular heart disease) were fitted for emergent initiation and for any early (30-day) complication, with a likelihood-ratio test of season as a whole; 3-month mortality (13 events) was described by season and analyzed with a season-only Firth model. Catheter dysfunction was analyzed among catheter-exposed patients, followed by sensitivity analyses. **Results**: A total of 58.3% of patients started dialysis emergently and 41.7% in a planned manner; only 11.3% had an arteriovenous fistula as initial vascular access. No significant seasonal differences were found in initiation type, indications, or the frequency of any early complication (161/314, 51.3%; *p* = 0.090). In the adjusted complication model, spring versus summer gave OR = 2.33 (95% CI: 1.23–4.41, *p* = 0.009; Holm-adjusted *p* = 0.028 across the three season contrasts), but the omnibus test of seasons was not significant (*p* = 0.069), and spring did not differ significantly from autumn or winter. Among catheter-exposed patients, catheter dysfunction occurred in 31.4% (22/70) in the spring and 16.5% (13/79) in the summer (*p* = 0.035; four-season omnibus *p* = 0.191); this contrast was not significant after excluding initiations from February to December 2023 (*p* = 0.111) or when the season was assigned at the midpoint of the risk window (*p* = 0.114). Season was not associated with emergent initiation (omnibus *p* = 0.230) or with 3-month mortality (Firth OR for spring versus summer 1.12, 95% CI: 0.28–4.48). **Conclusions**: Hemodialysis initiation in Adana was not associated with a winter-peak pattern in initiation counts or blood pressure. Spring initiation was associated with a higher frequency of early complications, mainly catheter dysfunction, but this exploratory single-center finding was not supported by the four-season omnibus tests and was sensitive to analytic choices, and none of the 33 seasonal omnibus tests remained significant after Holm correction. It is hypothesis-generating only and does not support a seasonal practice recommendation; confirmation in larger, multicenter, prospective studies is needed.

## 1. Introduction

Chronic kidney disease (CKD) is a major public health problem affecting approximately 10% of the adult population worldwide, with a steadily increasing prevalence; hemodialysis remains the most commonly used renal replacement modality among patients progressing to end-stage kidney disease (ESKD). According to the Turkish Society of Nephrology’s 2024 national registry report, the number of patients receiving renal replacement therapy in Türkiye reached 92,461, with an ESKD prevalence of 1079.3 and incidence of 164 per million population; 70.4% of patients were treated with hemodialysis, 25.4% with renal transplantation, and 4.2% with peritoneal dialysis, and diabetes mellitus was identified as the most common cause of ESKD [1].

The seasonal distribution of hemodialysis initiation has attracted growing interest in the nephrology literature. Studies from Japan—a single-center study [2] and a multicenter dialysis cohort [3]—as well as an international registry analysis [4] and national registry data [5]—have reported seasonal variation in hemodialysis initiation, in the transition to renal replacement therapy, or in related outcomes, with the Japanese reports describing a winter peak in initiation and a nadir in the summer [2,5]. This winter-peak pattern has been suggested to be related to a seasonal increase in volume overload and cardiac decompensation, particularly among elderly patients [2,3]. The MONDO registry study reported seasonal fluctuations in mortality and clinical indicators among hemodialysis patients [4]. However, the majority of these studies have been conducted in temperate or cold climate regions, and data from regions with Mediterranean/subtropical climates, such as Adana, our own study region, remain limited.

The mode of dialysis initiation (planned or emergent) and the type of initial vascular access are well known to play a determining role in early morbidity and mortality. The CHOICE study demonstrated that late nephrology referral significantly increases the likelihood of catheter use at hemodialysis initiation and prolongs catheter dependence [6]. Similarly, unplanned dialysis initiation and catheter-based access have been reported to be associated with increased hospitalization, infection, vascular access-related complications, and higher mortality [7,8]. Although vascular access guidance and quality initiatives emphasize arteriovenous fistula (AVF) creation prior to hemodialysis initiation [9], real-world data show that AVF utilization rates often remain below target levels [10].

Few studies have jointly examined the seasonal timing of hemodialysis initiation together with the mode of initiation and, in particular, early vascular access complications (catheter dysfunction, infection, and thrombosis) in a region with warm/subtropical climate characteristics. In this study, we retrospectively analyzed data from patients who started chronic hemodialysis at Adana City Training and Research Hospital, Department of Nephrology, between 2022 and 2025, aiming to evaluate the relationship between the season of hemodialysis initiation and the mode of initiation, indications, and early outcomes (complications and mortality).

## 2. Materials and Methods

### 2.1. Study Design

This study was designed as a single-center, retrospective, observational cohort study conducted at the Department of Nephrology, Adana City Training and Research Hospital. The study population consisted of consecutive patients who started chronic hemodialysis between 2022 and 2025. The parameters evaluated and their timing of assessment were as follows:

Demographic and clinical data (age, sex, comorbidities, and cause of primary renal disease) were obtained from patient records, based on documentation at the time of the first hemodialysis session. Laboratory parameters (creatinine, urea, eGFR, potassium, sodium, hemoglobin, albumin, pH, HCO3, and pro-BNP), blood pressure (systolic/diastolic), vascular access type, components of the emergency HD indication, and uremic symptoms were all recorded at a single time point, namely the clinical and biochemical assessment performed immediately before/at the first hemodialysis session (pre-dialysis baseline). Early complications (catheter infection, catheter dysfunction, AV fistula thrombosis, intradialytic hypotension, and other) were recorded in a single multi-category complication field, in which more than one complication could be recorded per patient, and refer to the first 30 days after hemodialysis initiation; hospitalization, ICU admission, and sepsis refer to the first 90 days. The complication field was blank or coded as unknown in 5 patients (spring 2, summer 1, and autumn 2), who were excluded from all complication analyses (*n* = 314). In three patients who started with an AVF, an erroneous catheter dysfunction code identified on review by the authors was removed. Mortality was defined as death within 1 month, 3 months, and 1 year of hemodialysis initiation (cumulative all-cause mortality up to each time point). For patients who died, the dataset holds the month of death (median 7.5 months, Q1–Q3 2–9, among the 36 deaths within one year of initiation); for survivors, the follow-up field holds a nominal value of 12 months (one patient: 22 months) rather than an observed time to last contact. Exact dates of death or last contact, and therefore true person-time and censoring times, were not available. The data lock was in July 2026 (vital status last updated in July 2026). The season of hemodialysis initiation was assigned using standard meteorological definitions for the Northern Hemisphere (spring: March–May; summer: June–August; autumn: September–November; winter: December–February), based on the calendar date of the first hemodialysis session; we recognize that these fixed calendar boundaries may not precisely track local climatic transitions in a subtropical setting such as Adana (see Limitations). Because winter spans two calendar years, December initiations were assigned to the calendar year in which dialysis started (December: 2022, *n* = 11; 2023, *n* = 7; 2024, *n* = 4; 2025, *n* = 2), so that “winter” of a given calendar year comprises January–February and December of that same year. The composite “cardiovascular disease” covariate used in the multivariable models denotes coronary artery disease and/or valvular heart disease, recorded in a single comorbidity field (171/319 patients, 53.6%); congestive heart failure, peripheral arterial disease, and stroke were recorded separately, are reported in the Results and were not part of this covariate. Planned versus emergent initiation was recorded in the clinical database as a categorical classification made by the treating team at the first session; no explicit algorithm is stored in the dataset, and its reliability was not assessed. The classification is not determined by access type: of the 133 planned initiations, 35 started with an AVF, 28 with a tunneled catheter, and 70 with a temporary catheter. Nor does it coincide with the presence of a documented “emergency HD indication” (volume overload, metabolic acidosis, hyperkalemia, or uremic symptoms): 47 planned initiations had a documented emergency indication, whereas 10 emergent initiations had none, which is why 223 patients had an emergency indication but only 186 were classified as emergent. Planned initiation therefore denotes the clinical impression of a non-urgent start rather than a start from a scheduled outpatient vascular access, and the results for Model 1 should be read with this in mind (see Limitations). Catheter dysfunction denotes inadequate blood flow through the catheter as recorded by the treating team in the complication field; no objective flow threshold and no mechanism (thrombosis, fibrin sheath, and malposition) were recorded. An indicator of catheter exchange due to dysfunction, derived during data cleaning, is identical to the dysfunction flag; the dataset therefore cannot distinguish dysfunction-driven exchanges from routine conversions of temporary to tunneled catheters. The insertion site was not recorded directly; in our unit, patients who are hospitalized at the start of dialysis receive femoral catheters and outpatients jugular catheters, so a recorded hospital stay at initiation (length-of-stay field > 0 days; 247 of 278 catheter-exposed patients) was used as a proxy for femoral insertion (31 patients as proxy for jugular insertion). The side, operator, and lock solution were not recorded. Catheter infection was defined clinically (fever, exit-site or tunnel-tract inflammation, or bacteremia temporally related to catheter use, per treating-physician documentation); microbiological confirmation was frequently unavailable and was not required. These definitions describe how the variables are recorded in the database; they were not re-adjudicated against the clinical charts. Pro-BNP values were collected but were missing for a substantial proportion of patients (180/319, 56.4%) and were therefore not included in the comparative statistical analyses. This missingness was not random with respect to clinical presentation: pro-BNP was measured in 57.2% of patients with documented volume overload at presentation versus only 25.9% of patients without it (Fisher’s exact test, *p* < 0.001, OR = 3.83), consistent with pro-BNP having been ordered selectively when volume overload or heart failure was clinically suspected rather than as a routine baseline test; this pattern of informative, indication-driven missingness is a further reason it was not used in the comparative analyses.

### 2.2. Inclusion and Exclusion Criteria

Inclusion Criteria:

Inclusion criteria were as follows: initiation of permanent (chronic) hemodialysis at our institution between 2022 and 2025; age 18 years or older at the time of initiation; and sufficiently complete demographic, clinical, and laboratory data for analysis. Exclusion criteria were as follows: temporary/acute dialysis for acute kidney injury (outside the chronic dialysis program); age younger than 18 years at initiation; and incomplete data insufficient for analysis.

A total of 365 patients who started chronic hemodialysis at our institution during the study period were initially screened for eligibility; 46 patients were excluded because of insufficient/incomplete demographic, clinical, or laboratory data, leaving 319 patients who met the inclusion criteria for analysis (Figure S1). The screening records did not document other reasons for exclusion; whether patients with acute kidney injury or aged <18 years had already been removed before the pool of 365 was assembled, as well as the breakdown of the 46 exclusions by variable, cannot be reconstructed (see Limitations). The database also does not record how chronicity (end-stage kidney disease rather than acute kidney injury requiring dialysis) was established for patients who started emergently. Analyses of complications included 314 patients with known complication status, as well as the fixed-window 1-year mortality analysis of 298 patients (Figure S1).

### 2.3. Objectives

The primary objective of the study was to evaluate whether the season of hemodialysis initiation (spring/summer/autumn/winter) is associated with the mode of dialysis initiation (planned vs. emergent) and related early outcomes (complications and early mortality). Secondary objectives included a descriptive comparison of patients’ general clinical profile by season, the distribution of monthly/yearly initiation counts, the distribution of vascular access type, and the seasonal distribution of catheter-related complications.

### 2.4. Statistical Analysis

The distribution of continuous variables was assessed using the Shapiro–Wilk test; continuous variables are presented as the median (Q1–Q3) and categorical variables as *n* (%). Chi-square tests (Fisher’s exact test where appropriate) were used for categorical variables and the Kruskal–Wallis test for continuous variables in seasonal comparisons. Multivariable logistic regression models were fitted for emergent initiation (Model 1; *n* = 319) and for any early complication (Model 2; *n* = 314 patients with known complication status), each adjusted for age, sex, diabetes, hypertension, and cardiovascular disease (coronary artery and/or valvular heart disease). These covariates were chosen on clinical grounds, without univariable screening. Initiation type and vascular access type were not covariates (initiation type is the outcome of Model 1; access type lies on the pathway to catheter-related outcomes). The models were not specified in a registered protocol, and summer was used as the reference season because it had the lowest observed frequency of complications; this choice was made after the descriptive pattern had been seen, and the spring-versus-summer contrast is therefore exploratory. Season as a whole was tested with a likelihood-ratio test comparing models with and without the three season indicators; all six pairwise season contrasts, alternative reference categories, and models additionally adjusted for calendar year are reported (Section 3.5 and Section 3.7, Supplementary Table S2). Three-month mortality (13 events) was described by season and analyzed with a Firth penalized logistic regression containing season alone (profile-likelihood confidence intervals) because a multivariable model with this few events is unstable. Catheter dysfunction, which can only occur in patients with a catheter in place, was analyzed as a primary comparison among catheter-exposed patients (temporary or tunneled catheter as initial access, with known outcome); sensitivity analyses addressed the denominator, exposure misclassification at season boundaries (exclusion of patients who started within 30 days before a season boundary; season assigned at the midpoint of the 30-day risk window), calendar year, and the period February–December 2023. Multiplicity was handled with Holm’s step-down procedure applied (i) to the three season-versus-summer contrasts of Model 2 and (ii) to the family of 33 seasonal omnibus tests reported in the Results. Analyses were complete-case: a patient was included in a model only if every variable in it was available (Model 1: *n* = 319; Model 2: *n* = 314). Changes in the proportion of emergent initiation across calendar years were examined with a chi-square test and a Cochran–Armitage trend test. Statistical significance was set at *p* < 0.05 (two-sided). The sample size was not based on an a priori power calculation; all eligible patients who met the inclusion criteria during the study period (2022–2025) were analyzed. Analyses were performed using Python (version 3.11; pandas 3.0.2, scipy 1.17.1, numpy 2.4.4, and openpyxl 3.1.5), with logistic and Firth regression models fitted by maximum likelihood (Newton–Raphson algorithm); the complete analysis script is provided as a Supplementary Code File.

### 2.5. Use of Artificial Intelligence

Generative AI (Claude, Anthropic, and Claude Sonnet 5) was used to assist with data cleaning and quality control of the raw dataset, to perform descriptive and inferential statistical analyses (chi-square, Fisher’s exact, Kruskal–Wallis tests, and logistic and Firth regression), and to generate tables and figures, including all analyses added during revision. In the course of the revision, the coding of every variable was re-audited against the source dataset; this identified two coding errors in the multivariable analysis as originally reported (the cardiovascular covariate and the handling of patients with unknown complication status), which were corrected, and the originally reported estimates were first reproduced exactly (Supplementary Table S1). All AI-assisted outputs were reviewed by the authors, who cross-checked reported statistics against the source dataset and take full responsibility for the accuracy and content of the manuscript.

## 3. Results

### 3.1. Patient Characteristics

A total of 319 patients who started chronic hemodialysis at our institution between 2022 and 2025 were included in the study. The median age was 62 years (Q1–Q3: 51–70), and 62.4% (*n* = 199) were male. The most common comorbidities were hypertension (83.4%), coronary artery and/or valvular heart disease (53.6%), diabetes mellitus (51.1%), and congestive heart failure (32.7%). While 58.3% (*n* = 186) of patients started dialysis under emergent conditions, only 41.7% (*n* = 133) started in a planned manner. Notably, only 11.3% (*n* = 36) of patients had an arteriovenous fistula (AVF) as their initial vascular access; the vast majority (79.6%, *n* = 254) started dialysis with a temporary catheter (Table 1).

### 3.2. Distribution by Year and Month

The number of hemodialysis initiations ranged from 70 to 92 per year (2022: *n* = 81, 2023: *n* = 92, 2024: *n* = 76, 2025: *n* = 70), with no clear increasing or decreasing trend across years. The monthly distribution showed the highest number of initiations in October (*n* = 35) and July (*n* = 34), and the lowest in September (*n* = 18) (Figure 1).

The year × season cross-tabulation showed that the seasonal distribution was not homogeneous across years (χ^2^ *p* = 0.044); notably, spring initiations markedly increased in 2023 (*n* = 30) (Table 2a).

In addition, the proportion of emergent initiation was lowest in 2025 (47.1%) compared with 59.3–64.1% in 2022–2024 (Table 2b). The overall difference across the four years was not statistically significant (chi-square *p* = 0.168; Cochran–Armitage trend test *p* = 0.126); a post hoc comparison of 2025 with 2022–2024 combined gave *p* = 0.039 (OR = 0.56), which is exploratory. The decline in 2025 is therefore a descriptive observation that does not by itself establish a temporal trend.

### 3.3. Seasonal Distribution of Initiation Type and Indications

Patient numbers were relatively balanced across seasons (spring *n* = 79, summer *n* = 88, autumn *n* = 82, and winter *n* = 70). The rate of emergent initiation was highest in the winter (65.7%) and pring (63.3%) and lowest in the summer (50.0%) (Figure 2); however, this difference did not reach statistical significance (*p* = 0.168). Similarly, no statistically significant seasonal differences were observed for vascular access type, components of the emergency HD indication (volume overload, metabolic acidosis, and hyperkalemia), the overall frequency of early complications (161/314, 51.3%; *p* = 0.090), or any of its components (catheter dysfunction, catheter infection, AV fistula thrombosis, and intradialytic hypotension; all *p* > 0.05) (Table 3). Catheter infection was the most frequent component (98/314, 31.2%), followed by catheter dysfunction (66/314, 21.0%), whereas AV fistula thrombosis and intradialytic hypotension each occurred in 29/314 patients (9.2%).

### 3.4. Seasonal Mortality and Early Events

The 1-month, 3-month, and 1-year mortality rates for the overall cohort are presented in Table 1. When compared by season, 1-year mortality was numerically higher in the autumn and winter, although this difference was not statistically significant (Table 4, Figure 3). No significant seasonal differences were observed for hospitalization, ICU admission, or sepsis within the first 90 days (Table 4).

### 3.5. Multivariable Analysis

In the multivariable logistic regression model for emergent initiation (Model 1; *n* = 319), adjusted for age, sex, diabetes, hypertension, and cardiovascular disease (coronary artery and/or valvular heart disease), season had no significant effect (omnibus likelihood-ratio test *p* = 0.230; spring versus summer OR = 1.68, 95% CI: 0.89–3.15, *p* = 0.108; winter versus summer OR = 1.82, 95% CI: 0.94–3.52, *p* = 0.076). Cardiovascular disease was not significantly associated with emergent initiation (OR = 0.65, 95% CI: 0.39–1.10, *p* = 0.110) (Table 5).

In the model for any early complication (Model 2; *n* = 314 patients with known complication status), spring initiation was associated with higher odds compared with summer (OR = 2.33, 95% CI: 1.23–4.41, *p* = 0.009; Holm-adjusted *p* = 0.028 for the three season-versus-summer contrasts), whereas autumn (OR = 1.39, 95% CI: 0.75–2.59, *p* = 0.298) and winter (OR = 1.62, 95% CI: 0.85–3.10, *p* = 0.143) did not differ significantly from summer. However, the omnibus likelihood-ratio test of season was not significant (*p* = 0.069), and spring did not differ significantly from autumn (OR = 1.68, 95% CI: 0.87–3.21, *p* = 0.120) or winter (OR = 1.44, 95% CI: 0.74–2.80, *p* = 0.289) (all contrasts: Supplementary Table S2). The crude frequencies of any complication were 62.3% in the spring, 42.5% in the summer, 50.0% in the autumn, and 51.4% in the winter, a crude risk ratio of 1.47 for spring versus summer; because the outcome affects more than half of patients, the odds ratio overstates the relative risk and should not be read as such. Catheter dysfunction was analyzed as a primary comparison among catheter-exposed patients with a known outcome (*n* = 278): it occurred in 22/70 (31.4%) spring initiations and 13/79 (16.5%) summer initiations (Fisher’s exact test *p* = 0.035, OR = 2.33; crude risk ratio 1.91), with 22.1% in the autumn and 23.0% in the winter (four-season omnibus *p* = 0.191; Table 3). By initial catheter type, dysfunction occurred in 58/249 temporary-catheter patients (spring 21/63, 33.3%; summer 11/72, 15.3%; autumn 13/58; winter 13/56; omnibus *p* = 0.104) and in 6/29 tunneled-catheter patients (1/7, 2/7, 2/10, and 1/5 across the four seasons), a group too small for a seasonal comparison (Table 3). The corresponding unrestricted figures were 23/77 (29.9%) versus 14/87 (16.1%; *p* = 0.041). The robustness of these contrasts is examined in Section 3.7.

Because only 13 deaths occurred within 3 months (spring 4/79, summer 4/88, autumn 3/82, and winter 2/70), a multivariable mortality model was not fitted. In a Firth penalized logistic regression containing season alone, the odds of 3-month death did not differ significantly from the summer in the spring (OR = 1.12, 95% CI: 0.28–4.48, *p* = 0.869), autumn (OR = 0.83, 95% CI: 0.18–3.50, *p* = 0.794), or winter (OR = 0.69, 95% CI: 0.12–3.19, *p* = 0.634; Table 5); the wide confidence intervals mean that the analysis is inconclusive rather than evidence of no association.

### 3.6. General Demographic and Laboratory Profile

In the seasonal comparison of general demographic and laboratory characteristics, no significant differences were found for age, sex distribution, comorbidities, or most laboratory parameters (creatinine, urea, eGFR, potassium, hemoglobin, albumin, pH, and HCO3). Only the serum sodium level showed a nominally significant, clinically small seasonal difference (Table 6; median 136 mEq/L in the spring, 137 in the summer, 138 in the autumn, and 137 in the winter; unadjusted *p* = 0.026). This does not survive correction for multiplicity (Holm-adjusted *p* = 0.858 across 33 seasonal tests) and is most likely a chance finding. The lowest sodium was observed in the spring rather than the summer, so the pattern does not fit a perspiration-loss explanation, and we are not aware of evidence in incident hemodialysis patients that would support one (one previously cited study concerns serum sodium in peritoneal dialysis patients [11], and another is about serum potassium in hemodialysis patients [12]). The difference should therefore not be interpreted further.

In addition, systolic and diastolic blood pressure values also showed no significant seasonal differences (Table 6). Notably, the observed pattern—lowest in the winter and highest in the spring/autumn—is the reverse of the classic “winter blood pressure peak” pattern frequently reported in the literature. Similarly, the distribution of hemodialysis initiation counts across seasons did not deviate significantly from a uniform distribution (chi-square goodness-of-fit test, χ^2^ = 2.12, *p* = 0.549). With 319 initiations and 3 degrees of freedom, this test has 80% power only for a fairly large departure from uniformity (Cohen’s w ≈ 0.185, equivalent to, for example, a winter share of about 33% instead of the 25% expected), so moderate seasonal differences in initiation counts cannot be excluded.

### 3.7. Sensitivity Analyses and Multiplicity

To examine how robust the spring-versus-summer difference in catheter dysfunction is, we performed several sensitivity analyses among catheter-exposed patients (Table 7). Adjusted for age, sex, diabetes, hypertension, and cardiovascular disease, the spring-versus-summer odds ratio was 2.30 (95% CI: 1.04–5.07, *p* = 0.039; omnibus *p* = 0.222) and became 3.25 (95% CI: 1.35–7.85, *p* = 0.009; omnibus *p* = 0.051) after additionally adjusting for calendar year. Calendar-year adjustment likewise strengthened the any-complication model (Model 2: spring versus summer OR = 2.62, 95% CI: 1.33–5.18, *p* = 0.005; omnibus *p* = 0.041; the odds of any complication were lower in 2024 and 2025 than in 2022, ORs 0.26 and 0.27), whereas the emergent-initiation model was essentially unchanged (omnibus *p* = 0.305). Excluding patients who started within 30 days before a season boundary (90 catheter-exposed patients excluded) left the contrast significant (15/46, 32.6% versus 8/57, 14.0%; OR = 2.96, *p* = 0.032), whereas assigning season by the midpoint of the 30-day risk window did not (20/70, 28.6% versus 13/77, 16.9%; OR = 1.97, *p* = 0.114). Excluding the 85 initiations from February to December 2023 (spring 2023: *n* = 30) left the point estimate similar but non-significant (14/41, 34.1% versus 12/61, 19.7%; OR = 2.12, *p* = 0.111; adjusted OR = 2.29, 95% CI: 0.91–5.73, *p* = 0.077, *n* = 203).

Spring catheter dysfunction was numerically higher than summer in each of the four calendar years (Table 7), but the numbers per year are small, and no single-year contrast was significant. Notably, the spring 2023 rate (8/29, 27.6%) was not higher than that of spring 2022 (6/12, 50.0%) or spring 2024 (6/18, 33.3%), so the 2023 spring cluster does not by itself account for the overall contrast. Five patients with an AVF as initial access carried a catheter dysfunction code in the original database. For three of them (autumn, winter, and one spring patient, none with a recorded change of access), the authors confirmed that no catheter dysfunction had occurred, and the code was removed; the other two (spring 1 and summer 1) had a bridging catheter placed within 30 days, in which the dysfunction occurred, and they are included in the alternative denominator (Table 7). The three corrections did not change any catheter-exposed analysis. Baseline hemoglobin did not differ between catheter-exposed patients with and without catheter dysfunction (median 8.95 versus 8.9 g/dL, *p* = 0.98).

Insertion site was approximated by hospital stay at initiation (assumed femoral; otherwise assumed jugular; see Methods Section 2.1). The proxy classified 247 of the 278 catheter-exposed patients as femoral and 31 as jugular, and its distribution did not differ across seasons (*p* = 0.736). Catheter dysfunction occurred in 60/247 (24.3%) proxy-femoral and 4/31 (12.9%) proxy-jugular patients (Fisher’s exact test *p* = 0.181). Among proxy-femoral patients, dysfunction occurred in 21/63 (33.3%) in the spring and 13/68 (19.1%) in the summer (OR = 2.12, *p* = 0.075); the proxy-jugular group (spring 1/7 and summer 0/11) was too small for comparison. Adding the proxy to the adjusted model changed the spring-versus-summer estimate little (OR = 2.25, 95% CI: 1.01–4.98, *p* = 0.046; omnibus *p* = 0.252; proxy OR = 2.15, 95% CI: 0.71–6.50, *p* = 0.176). Because the proxy is indirect and highly unbalanced, it cannot exclude confounding by insertion site.

Regarding multiplicity, the Holm-adjusted *p* values for the three season-versus-summer contrasts of Model 2 were 0.028 (spring), 0.298 (autumn), and 0.287 (winter). Across the family of 33 seasonal omnibus tests reported in Table 3 and Table 4, and Table 6, the smallest unadjusted *p* value was 0.026 (serum sodium), and the smallest Holm-adjusted *p* value was 0.858; none of the omnibus seasonal tests therefore remained significant after correction.

## 4. Discussion

Our findings point to two main themes: (1) the classic “winter peak” pattern (initiation counts, blood pressure) was not observed in our cohort, and for some variables, the trend was even in the opposite direction; (2) spring initiations had a higher frequency of early complications than summer initiations (crude 62.3% versus 42.5%; adjusted OR = 2.33, 95% CI: 1.23–4.41), mainly reflecting more catheter dysfunction (31.4% versus 16.5% among catheter-exposed patients). However, the omnibus tests of season were not significant (any complication *p* = 0.069; catheter dysfunction *p* = 0.191), the catheter dysfunction contrast lost significance in some sensitivity analyses, and no seasonal omnibus test remained significant after correction for multiplicity. These observations are therefore hypothesis-generating; because no climatic, workload, or staffing variable was measured, they cannot indicate by which mechanism, if any, season might act.

### 4.1. Association Between Spring Initiation and Catheter Dysfunction

Before interpreting this association, it should be reconciled with the overall (four-season) comparisons, which were not significant: any early complication, with *p* = 0.090 (Table 3; omnibus likelihood-ratio test in the adjusted model, *p* = 0.069), and catheter dysfunction among catheter-exposed patients, with *p* = 0.191. The significant spring-versus-summer contrast arises when summer is the reference category; summer was chosen after the descriptive pattern had been seen because it had the lowest observed complication frequency, so this contrast is exploratory rather than prespecified. Spring did not differ significantly from autumn or winter (Supplementary Table S2). The calendar year was non-homogeneously distributed across seasons (*p* = 0.044; Table 2a), with a disproportionate number of spring initiations in 2023. Adjusting for the calendar year did not remove the spring contrast but strengthened it (any complication: OR = 2.62, omnibus *p* = 0.041; catheter dysfunction: OR = 3.25, omnibus *p* = 0.051), partly because the odds of complications fell markedly over calendar time (ORs 0.26 for 2024 and 0.27 for 2025 versus 2022), which indicates a secular decline in complications unrelated to season; season and calendar year are partly intertwined in this cohort, and secular changes in catheter care practice, staffing, or case mix cannot be excluded as contributors.

Catheter dysfunction can by definition only occur in patients with a catheter in place, so the primary analysis was restricted to catheter-exposed patients (278 of the 314 patients with known complication status, 88.5%): dysfunction occurred in 22/70 (31.4%) spring initiations versus 13/79 (16.5%) summer initiations (Fisher’s exact test, *p* = 0.035, OR = 2.33; crude risk ratio 1.91), compared with 23/77 (29.9%) versus 14/87 (16.1%) when all patients were included (*p* = 0.041). Stratified by catheter type, dysfunction occurred in 58/249 (23.3%) temporary-catheter patients and 6/29 (20.7%) tunneled-catheter patients; the spring-versus-summer pattern was present within the temporary-catheter subgroup (spring 21/63, 33.3%, versus summer 11/72, 15.3%), whereas the tunneled-catheter subgroup was too small (5–10 patients per season) for a meaningful seasonal comparison. The sensitivity analyses (Section 3.7, Table 7) show a consistent direction but statistical support that depends on the analytic choice: *p* between 0.009 and 0.039 in the boundary-exclusion, alternative-denominator, and adjusted analyses (with and without calendar year) but *p* = 0.111 and 0.114 when excluding February–December 2023 or assigning the season by the midpoint of the risk window. We therefore regard the spring–catheter dysfunction association as hypothesis-generating rather than conclusive. A further limitation is that scheduled conversions of temporary to tunneled catheters cannot be separated from dysfunction-driven exchanges in the dataset; if seasonal differences in access to interventional or operating-room capacity influenced the timing of such conversions, they could contribute to an artifactual seasonal signal, and the insertion site, a major determinant of temporary catheter dysfunction, could be approximated only by a proxy (hospital stay at initiation; Section 3.7), while the side, operator, and lock solution were not recorded.

Spring 2023 contained 30 initiations, versus 13–21 in the other spring seasons (Table 2a), and followed the 6 February 2023 Kahramanmaraş earthquakes, which affected Adana and very severely affected Hatay. Patients’ place of residence, displacement, crush-related kidney injury, and disruption of predialysis care were not recorded, so we cannot determine whether any of the excess spring 2023 initiations, or the conditions under which catheters were placed and cared for during that period, were related to the earthquakes; this is raised only as a possible, untested contributor to the 2023 spring excess. The sensitivity analysis excluding February–December 2023 gave a similar point estimate (OR = 2.12 versus 2.33 in the primary analysis) with a wider confidence interval that no longer excluded no difference, and the spring 2023 catheter dysfunction rate was not higher than in spring 2022 or 2024, so the 2023 cluster does not by itself appear to explain the contrast; the loss of significance is, however, also expected from the loss of 85 of 319 initiations.

Previous studies have shown that hemodialysis initiation peaks in the winter, partly related to a seasonal increase in volume overload and cardiac decompensation [2,3]; in our cohort, a higher complication frequency was seen in the spring and was mostly due to catheter dysfunction rather than catheter infection or AV fistula thrombosis. Several explanations can be considered, and our data allow only a limited examination of them. First, seasonal hemoconcentration/dehydration could, in principle, raise the risk of catheter thrombosis/dysfunction; baseline hemoglobin was highest in the spring (median 9.2 versus 8.9 g/dL in the other seasons, Table 6) but did not differ between catheter-exposed patients with and without dysfunction (*p* = 0.98), and the hypothesis would also predict a similar or greater effect in the warmer summer months, when complications were least frequent. Second, seasonal fluctuations in hospital workload may affect the quality of catheter care; however, the highest monthly initiation counts occurred in July (*n* = 34) and October (*n* = 35) (Figure 1), when complication frequency was not high, which does not support a simple initiation-workload explanation, although hospital workload and staffing data were not collected. Third, regional exposures specific to Adana (e.g., pollen or dust) cannot be assessed with our data. Non-climatic explanations—catheter-specific factors (insertion site, operator, lock solution, and timing of conversion to tunneled catheters), secular changes in practice, the 2023 spring cluster discussed above, and chance—are at least as plausible. Our findings should therefore not be taken as evidence for any climate-specific mechanism.

Catheter dysfunction has been attributed to various factors including thrombus formation, fibrin sheath development, and catheter malposition; female sex, hypertension, and obesity have been reported as risk factors for thrombosis-related dysfunction [13]. As few studies have directly examined this seasonal relationship, our findings warrant confirmation in future prospective studies.

### 4.2. High Rate of Emergent Initiation and Low AVF Utilization

The finding that 58.3% of patients in our study started dialysis under emergent conditions, with only 11.3% having an AVF, suggests that late nephrology referral or inadequate predialysis preparation may be an important issue at our institution. Indeed, nearly all patients who started with an AVF (35/36, 97.2%) did so in a planned manner, whereas the majority of patients who started with a temporary catheter (184/254, 72.4%) were initiated emergently. This finding is consistent with the CHOICE study, which demonstrated that late nephrology referral increases catheter use [6]. Furthermore, unplanned initiation and catheter-based access have been associated with higher hospitalization, infection, and mortality rates [7,8], underscoring the need to prioritize early nephrology referral and predialysis vascular access planning in clinical practice. We note that the incident AVF proportion in a predominantly emergent cohort is not directly comparable with the prevalent-access targets of vascular access programs, and we therefore do not benchmark it against a numerical target.

In the adjusted model for emergent initiation, coronary artery and/or valvular heart disease (171/319 patients, 53.6%) was not significantly associated with emergent initiation (OR = 0.65, 95% CI: 0.39–1.10, *p* = 0.110). The point estimate points towards lower odds of emergent initiation, which would be compatible with closer cardiology and/or nephrology follow-up facilitating timely planning of dialysis, as suggested by a systematic review of 27 cohort studies (17,646 patients) in which early nephrology referral was associated with reduced mortality and hospitalization and better dialysis preparation, including timely creation of permanent vascular access [14]; however, the confidence interval includes no effect, follow-up intensity and referral timing were not measured, and the planned/emergent classification rests on clinical judgment of unknown reliability (see Limitations). We therefore draw no conclusion from this estimate.

### 4.3. Decline in Emergent Initiation over Time

The lower rate of emergent initiation in 2025 (47.1%) compared with 2022–2024 (59.3–64.1%) could reflect a gradual improvement in predialysis care processes at our institution, but this interpretation is tentative. The year × season chi-square test (*p* = 0.044) only shows that the distribution of seasons differed across years and says nothing about the proportion of planned versus emergent initiation; a separate test across years was not significant (*p* = 0.168), nor was a trend test (*p* = 0.126), and only a post hoc comparison of 2025 with earlier years reached *p* = 0.039. Possible explanations, which cannot be distinguished in this retrospective, descriptive design, include an increase in nephrology outpatient capacity, growing awareness of predialysis education programs, improvement in the primary care-to-nephrology referral chain, or institutional adjustments to dialysis bed and vascular access planning; confirming any of them would require a prospective study tracking predialysis referral records over time.

### 4.4. Unexpected Seasonal Pattern of Blood Pressure and Hemodialysis Initiation Counts

A strong and consistent body of literature indicates that blood pressure in the general population and in hemodialysis patients peaks in the winter and reaches its nadir in the summer; this pattern was first described by Argilés et al. [15], later confirmed together with overhydration by Sposito et al. [16], and reaffirmed in Usvyat et al.’s 5-year cohort study [17]. In our study, however, blood pressure was measured once, at or immediately before the first hemodialysis session, so the seasonal comparison is necessarily cross-sectional (different patients initiating in different seasons) rather than a within-patient, repeated-measures comparison as used in most of the studies cited above; our results therefore more precisely indicate no significant seasonal difference in baseline blood pressure at dialysis initiation, rather than an absence of blood pressure seasonality in the conventional longitudinal sense, and the two types of evidence are not directly interchangeable. With this caveat in mind, blood pressure values in our cohort showed no significant seasonal difference, and the observed trend (lowest in the winter and highest in the autumn/spring) was in fact the reverse of the classic pattern. This finding is not isolated: Fine reported no seasonal variation in blood pressure among hemodialysis patients at a North American center despite winter temperatures as low as −20 °C [18]. Furthermore, a multicenter study by Duranton et al. across five European cities demonstrated that blood pressure seasonality varies markedly with latitude and climate, weakening or disappearing in temperate/warm climate regions [19]. In this context, the fact that our study was conducted in Adana, a city with a Mediterranean/subtropical climate, is one possible reason why neither blood pressure nor hemodialysis initiation counts (χ^2^ = 2.12, *p* = 0.549) exhibited the expected winter-peak pattern; this is compatible with, but does not demonstrate, climate-dependent seasonality because no climatic variable was measured. In addition, most of our patients started emergently (58.3%), and 56.4% had volume overload at presentation, so single baseline blood pressure values largely reflect acute volume status rather than seasonal physiology.

### 4.5. Mortality Findings

The 1-month and 3-month mortality rates in our study were 1.6% and 4.1% (entire cohort, *n* = 319), respectively; 1-year mortality was 11.1% (33/298) among the patients included in the fixed-window analysis. These rates are generally consistent with incident hemodialysis cohorts reported in the literature. Because only 13 deaths occurred within 3 months, seasonal differences in mortality were assessed with crude proportions and a season-only Firth model, which did not detect a statistically significant association (Table 5), a result that should be interpreted in light of the very limited number of events (see Limitations). Although Japanese multicenter cohorts have reported that the season of hemodialysis initiation may be associated with subsequent cardiac events [3], we did not detect a statistically significant association between season and mortality in our cohort; given the limited power of this analysis, this should be read as an inconclusive result rather than as evidence that no such association exists.

A Kaplan–Meier analysis that retains late starters as censored observations was not feasible: the dataset contains neither dates of death or last contact nor an observed follow-up time for survivors (who carry a nominal 12 months), so there are no valid censoring times, and a survival curve would merely reproduce the crude one-year proportions. We therefore report fixed-window mortality only. Of the 36 deaths recorded within one year of initiation (month of death: median 7.5, Q1–Q3 2–9), 33 occurred among the 298 patients included in the fixed-window analysis and 3 among the 21 patients who started dialysis between 4 September and 4 December 2025 and were excluded from it. The data lock was in July 2026 (Methods Section 2.1), so patients who started up to July 2025 had at least 12 months of potential observation; the seven patients who started in August 2025 may have had slightly less, and the 12-month window is assumed complete for them. Excluding all initiations after 30 June 2025 (*n* = 284; 31 deaths) gave a similar result (season difference *p* = 0.70). One-year mortality is presented as a descriptive statistic only.

### 4.6. Strengths of the Study

This study has several notable strengths. First, the vast majority of the seasonal hemodialysis initiation literature is based on studies conducted in cold or temperate climate regions (Japan, North America, and Europe); our data from a warm/subtropical climate region fill an important gap in the literature. Second, our study does not merely fill a data gap but actively reveals an unexpected finding: the classic “winter peak” pattern (in both hemodialysis initiation counts and blood pressure) was not observed in our cohort, and for some variables, the trend was even in the opposite direction; this finding is consistent with, although does not by itself prove, the hypothesis that seasonal effects on dialysis-related outcomes may vary by climate and differ in mechanism from those described in cold/temperate settings; confirmation would require direct measurement of climatic exposures, which was beyond the scope of this retrospective analysis. Third, the higher frequency of early catheter dysfunction in the spring, reported here together with omnibus tests, alternative reference categories, calendar-year adjustment, and boundary and denominator sensitivity analyses, is a signal that we regard as hypothesis-generating and worth targeted confirmation in future prospective studies; it was not supported by the four-season omnibus tests and does not remain significant after correction for multiplicity, so it is not an immediately actionable practice change. Fourth, whereas most previous studies have focused on a single outcome (only mortality or only blood pressure), our study evaluated mode of initiation, vascular access, complications, mortality, and blood pressure within the same cohort, providing a more complete picture. Finally, the analysis code and a data dictionary are provided as Supplementary Files so that every reported estimate can be reproduced from the dataset.

### 4.7. Limitations

This study has several limitations. It is retrospective and single-center in design; therefore, causality cannot be established, and findings should be interpreted as associative only. Some variables contained missing or “unknown”-coded data, particularly evident in microbiological culture results and certain laboratory parameters (e.g., pro-BNP, which was consequently excluded from the comparative analyses). Of the 365 patients initially screened for eligibility during the study period, 46 (12.6%) were excluded because of insufficient/incomplete demographic, clinical, or laboratory data, leaving the final analytic cohort of 319 (Figure S1). All 46 exclusions were recorded as incomplete data; a breakdown by specific variable, as well as any exclusions for acute kidney injury or age that may have preceded the screening pool, were not documented and cannot be reconstructed retrospectively. The database also does not record how chronicity was established at an emergent start; patients who died early or recovered kidney function may therefore have been systematically excluded or included, which could bias comparisons across seasons. The per-variable extent of missing data among the 319 analyzed patients was as follows: core demographic and most laboratory variables (age, sex, creatinine, urea, eGFR, potassium, sodium, hemoglobin, pH, HCO3, diabetes, hypertension, and stroke) were complete; albumin and congestive heart failure status were each missing for 1 patient (0.3%); systolic and diastolic blood pressure were each missing for 5 patients (1.6%); the complication field was unknown for 5 patients (1.6%); and pro-BNP was missing for 180 patients (56.4%) and was excluded from the comparative analyses. Analyses were complete-case, as described in Methods Section 2.4. The outcome definitions describe how variables are recorded in the database and were not re-adjudicated against the charts. In three patients who started with an AVF, an erroneous catheter dysfunction code was identified and removed by the authors; two other AVF-start patients developed dysfunction in a bridging catheter. Of the 29 patients coded with AV fistula thrombosis, 22 had started with a catheter (19 of them with a recorded change of access within 30 days, compatible with fistula creation before the event but not verifiable); the coding of this component may therefore also contain errors. The specific causes of catheter dysfunction (thrombus, kinking, malposition, etc.) were not differentiated, no objective criterion (e.g., a blood-flow threshold) was recorded, scheduled conversions of temporary to tunneled catheters could not be separated from dysfunction-driven exchanges, and the insertion site was only approximated by a proxy (hospital stay at initiation, assumed femoral; otherwise assumed jugular), while the side, operator, and lock solution were not recorded. Planned versus emergent initiation reflects the treating team’s clinical classification of unknown reliability, which conflicts in part with the emergency-indication variable (47 planned starts with a documented emergency indication; 10 emergent starts without) and with access type (70 planned starts via a temporary catheter); this directly affects Model 1. Season was assigned according to the calendar date of the first hemodialysis session, whereas complications and other early events were ascertained over the following 30 or 90 days; a patient initiating near a seasonal boundary therefore carries part of their risk window into the following season, which blurs the exposure-outcome timing (examined in Section 3.7 but not eliminated). The reference season was chosen after the descriptive pattern had been seen. Calendar year was non-homogeneously distributed across seasons; adjustment for it was reported as a sensitivity analysis only, and the temporal coincidence of spring 2023 with the February 2023 earthquakes could not be examined because the place of residence and displacement were not recorded. No climatic, workload, or staffing variable was measured, so no mechanism can be tested, and blood pressure was measured once at initiation in a cohort in which most patients started emergently and more than half had volume overload. Follow-up time is nominal for survivors, and exact dates of death or last contact were unavailable, so time-to-event analyses were not possible, and 1-year mortality is descriptive only; the main power limitation concerns the small number of events for outcomes such as 1- and 3-month mortality (13 events for the 3-month endpoint), for which only descriptive and season-only Firth estimates are presented, with very wide confidence intervals. The goodness-of-fit test of initiation counts could detect only a fairly large departure from uniformity (Cohen’s w ≈ 0.185). Because many exploratory seasonal comparisons were performed, individually significant results should be interpreted with the possibility of false-positive findings in mind; after Holm correction across 33 omnibus seasonal tests, none remained significant, and the risk of type II error should likewise be considered for the non-significant comparisons. Finally, seasonal effects may be specific to the subtropical climate conditions of Adana province, and the generalizability of the findings to centers in different climate zones may be limited.

### 4.8. Clinical Implications and Recommendations for Future Research

Larger, multicenter prospective studies are needed to determine whether early catheter dysfunction varies by season, with prospective recording of the insertion site, side, operator, lock solution, planned conversions, and hospital workload and staffing and with direct climatic measurements; our data do not support a practice recommendation specific to spring initiations. Independently of season, the low AVF utilization rate and the high proportion of emergent starts indicate a need to strengthen predialysis vascular access planning at our institution.

## 5. Conclusions

In this single-center, retrospective, exploratory analysis, hemodialysis initiation in Adana, a region with a subtropical climate, did not exhibit the classic “winter peak” pattern (initiation counts and blood pressure) described in cold/temperate climate regions. Patients starting in the spring had a higher frequency of early complications than those starting in the summer, mainly attributable to catheter dysfunction (adjusted OR = 2.33 for any complication; 31.4% versus 16.5% for catheter dysfunction among catheter-exposed patients). However, the omnibus tests of season were not significant (*p* = 0.069 and *p* = 0.191), the catheter dysfunction contrast was sensitive to the exclusion of 2023 and to the assignment of season, summer as the reference was chosen after the data were seen, and none of the 33 seasonal omnibus tests remained significant after Holm correction (the spring-versus-summer contrast of the complication model had a Holm-adjusted *p* = 0.028 within its own family of three contrasts). These findings are hypothesis-generating only; they do not support seasonal practice recommendations, and confirmation in larger, prospective, multicenter studies with measured climatic, workload, and catheter-specific variables is needed.

## Figures and Tables

**Figure 1:**
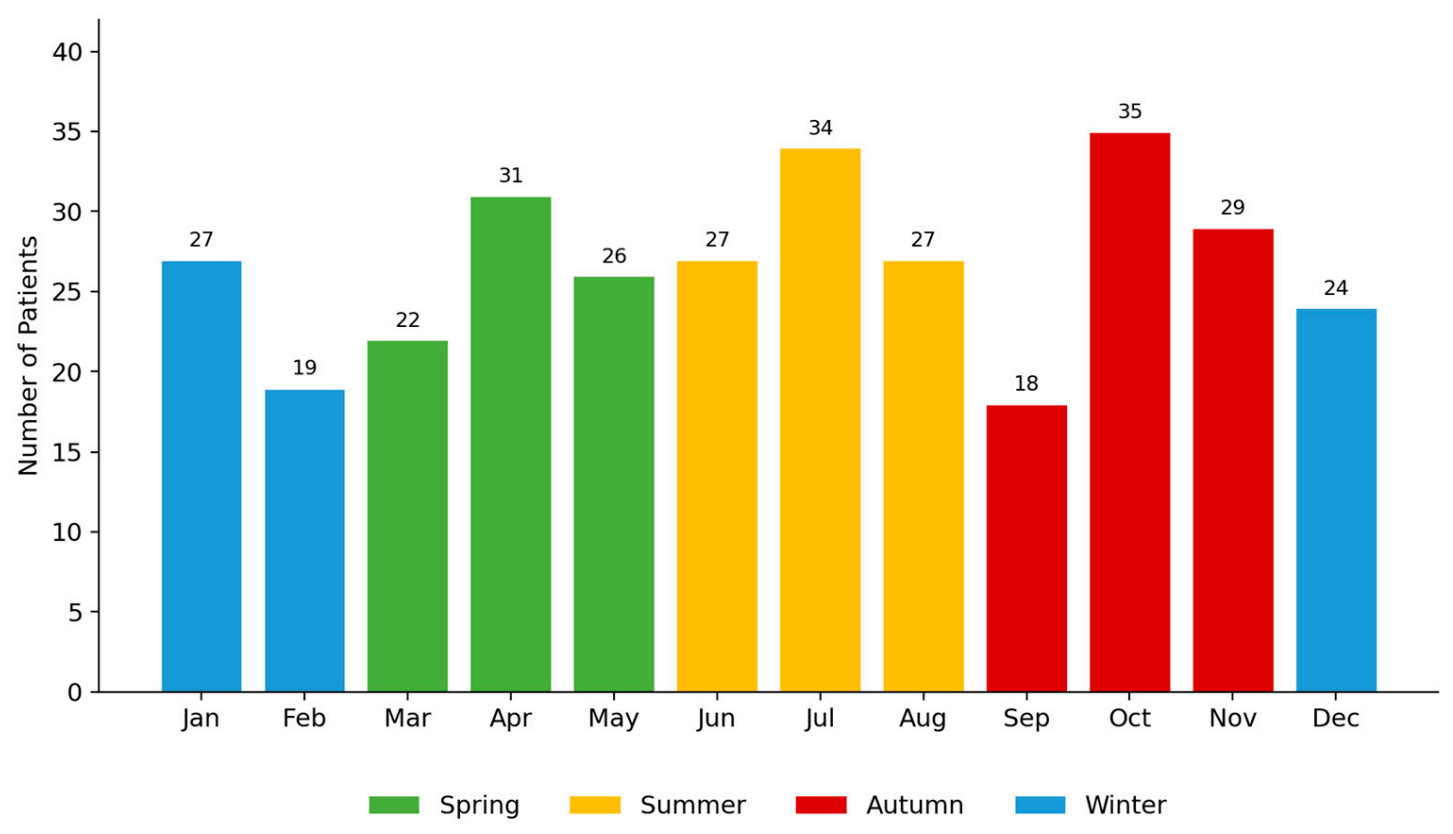
Monthly distribution of hemodialysis initiation (2022–2025, *n* = 319).

**Figure 2:**
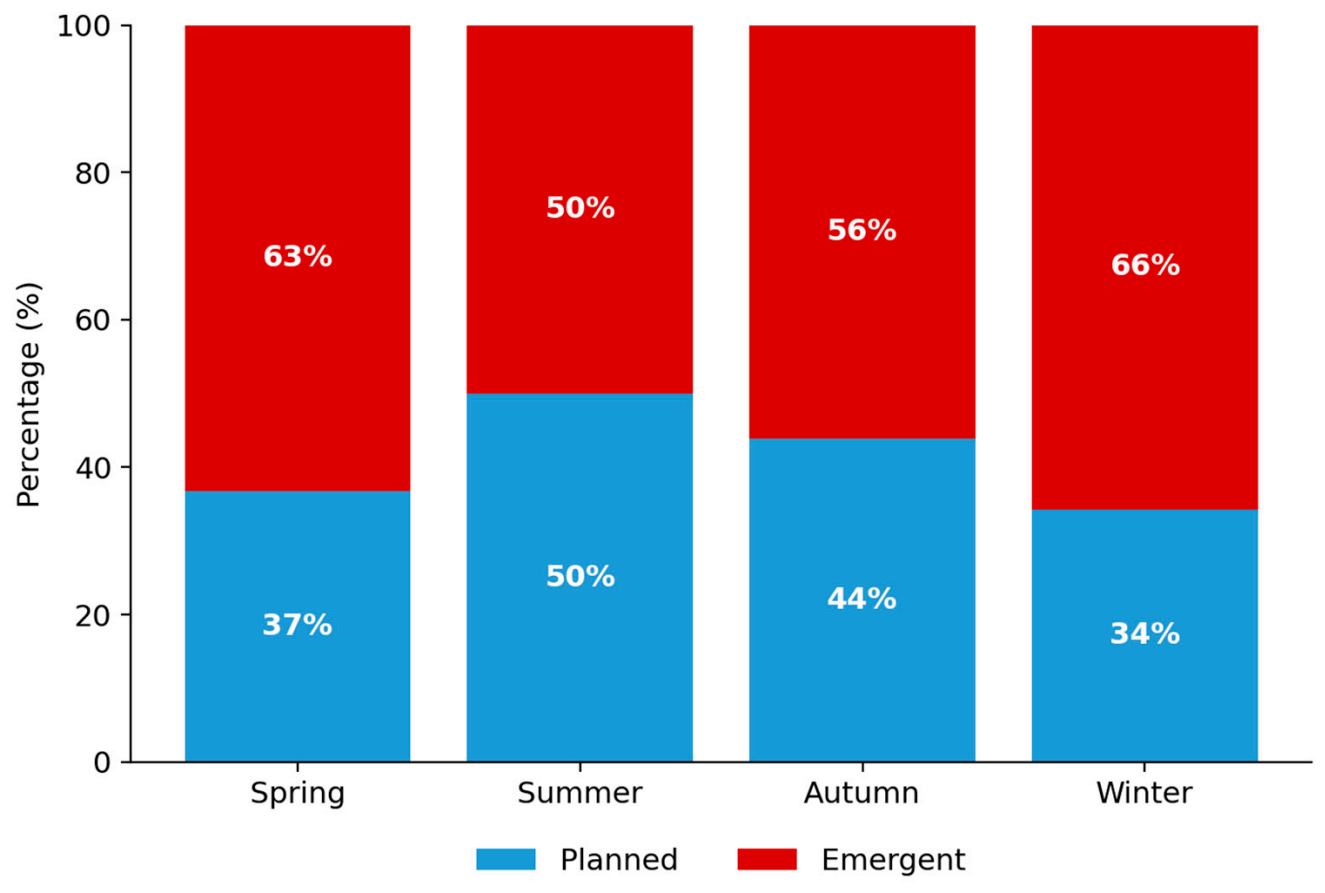
Distribution of initiation type by season (*n* = 319).

**Figure 3:**
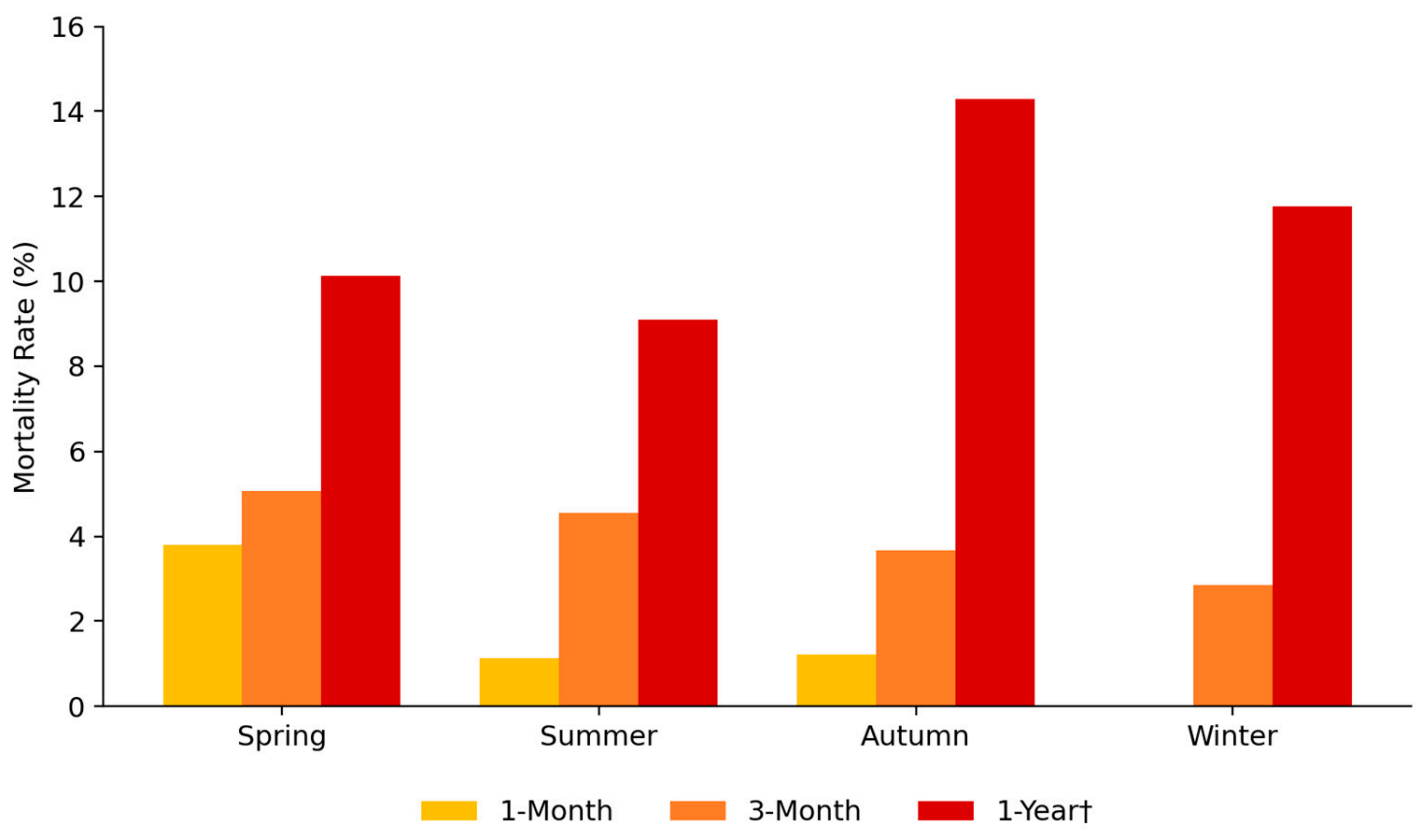
The 1-/3-month (*n* = 319) and 1-year (*n* = 298 †) mortality rates by season.

**Table 1:** General patient characteristics (*n* = 319).

Demographic Characteristics	
Age, median (Q1–Q3)	62 (51–70)
Sex, *n* (%)	
Male	199 (62.4%)
Female	120 (37.6%)
Comorbidities, *n* (%)	
Hypertension	266 (83.4%)
Diabetes Mellitus	163 (51.1%)
Congestive Heart Failure	104 (32.7%)
Peripheral Arterial Disease	39 (12.2%)
Chronic Lung Disease	37 (11.6%)
Stroke (CVA)	36 (11.3%)
Coronary Artery and/or Valvular Disease	171 (53.6%)
Initiation Type, *n* (%)	
Emergent	186 (58.3%)
Planned	133 (41.7%)
Initial Vascular Access, *n* (%)	
Temporary Catheter	254 (79.6%)
Arteriovenous Fistula (AVF)	36 (11.3%)
Tunneled Catheter	29 (9.1%)
Laboratory Findings	
Creatinine (mg/dL), median (Q1–Q3)	7.2 (6.1–9.1)
Urea (mg/dL), median (Q1–Q3)	187 (143–226)
eGFR (mL/min), median (Q1–Q3)	6.0 (5.0–8.0)
Potassium (mEq/L), median (Q1–Q3)	4.9 (4.3–5.5)
Sodium (mEq/L), median (Q1–Q3)	137 (134–139)
Hemoglobin (g/dL), median (Q1–Q3)	9.0 (8.0–10.0)
Albumin (g/dL), median (Q1–Q3)	3.3 (2.9–3.6)
pH, median (Q1–Q3)	7.3 (7.2–7.3)
HCO3 (mEq/L), median (Q1–Q3)	17.7 (14.0–21.0)
Mortality, *n* (%)	
1-Month Mortality	5 (1.6%)
3-Month Mortality	13 (4.1%)
1-Year Mortality	33 (11.1%) ^†^

Continuous variables are presented as the median (Q1–Q3). Categorical variables are presented as *n* (%). ^†^ The 1-year mortality was calculated among 298 patients not excluded for insufficient follow-up (see Methods Section 2.1 and Limitations).

**Table 2:** (**a**). Year × season cross-tabulation, *n*. (**b**) Distribution of initiation type by year.

(**a**)					
**Year**	**Spring**	**Summer**	**Autumn**	**Winter**	**Total**
2022	15	31	23	12	81
2023	30	20	17	25	92
2024	21	15	23	17	76
2025	13	22	19	16	70
Total	79	88	82	70	319
(**b**)					
**Year**	**2022 (*n* = 81)**	**2023 (*n* = 92)**	**2024 (*n* = 76)**	**2025 (*n* = 70)**	
Planned, *n* (%)	33 (40.7%)	33 (35.9%)	30 (39.5%)	37 (52.9%)	
Emergent, *n* (%)	48 (59.3%)	59 (64.1%)	46 (60.5%)	33 (47.1%)	

Chi-square test: *p* = 0.044. Winter comprises January–February and December of the calendar year of initiation.

**Table 3:** Seasonal distribution of initiation type, indications, and early complications.

Variable	Spring (*n* = 79)	Summer (*n* = 88)	Autumn (*n* = 82)	Winter (*n* = 70)	*p*
Initiation Type, *n* (%)					
Planned	29 (36.7%)	44 (50.0%)	36 (43.9%)	24 (34.3%)	0.168
Emergent	50 (63.3%)	44 (50.0%)	46 (56.1%)	46 (65.7%)	
Initial Vascular Access, *n* (%)					
AVF	7 (8.9%)	8 (9.1%)	12 (14.6%)	9 (12.9%)	0.721
Tunneled Catheter	7 (8.9%)	7 (8.0%)	10 (12.2%)	5 (7.1%)	
Temporary Catheter	65 (82.3%)	73 (83.0%)	60 (73.2%)	56 (80.0%)	
Components of Emergency HD Indication, *n* (%)					
Emergency HD Indication (present)	58 (73.4%)	58 (65.9%)	54 (65.9%)	53 (75.7%)	0.408
Volume Overload	46 (58.2%)	45 (51.1%)	46 (56.1%)	43 (61.4%)	0.610
Metabolic Acidosis	56 (70.9%)	53 (60.2%)	62 (75.6%)	50 (71.4%)	0.159
Hyperkalemia	27 (34.2%)	30 (34.1%)	35 (42.7%)	28 (40.0%)	0.587
Early Dialysis Complications within 30 Days, *n*/*N* (%)					
Any Complication	48/77 (62.3%)	37/87 (42.5%)	40/80 (50.0%)	36/70 (51.4%)	0.090
Catheter Dysfunction (all patients)	23/77 (29.9%)	14/87 (16.1%)	15/80 (18.8%)	14/70 (20.0%)	0.158
Catheter Dysfunction (catheter-exposed)	22/70 (31.4%)	13/79 (16.5%)	15/68 (22.1%)	14/61 (23.0%)	0.191
Temporary Catheter	21/63 (33.3%)	11/72 (15.3%)	13/58 (22.4%)	13/56 (23.2%)	0.104
Tunneled Catheter	1/7 (14.3%)	2/7 (28.6%)	2/10 (20.0%)	1/5 (20.0%)	—
Catheter Infection	26/77 (33.8%)	26/87 (29.9%)	25/80 (31.3%)	21/70 (30.0%)	0.950
AV Fistula Thrombosis	10/77 (13.0%)	7/87 (8.0%)	5/80 (6.3%)	7/70 (10.0%)	0.505
Intradialytic Hypotension	11/77 (14.3%)	5/87 (5.7%)	9/80 (11.3%)	4/70 (5.7%)	0.170

Categorical variables are presented as *n* (%) or, for complication rows, *n*/*N* (%); *p* values were calculated using the chi-square test. Complication rows exclude 5 patients with unknown complication status (spring 2, summer 1, and autumn 2); the components are not mutually exclusive, and one further complication coded “other” (winter) is not shown. “Catheter-exposed” rows include only patients whose initial access was a temporary or tunneled catheter and whose outcome was known.

**Table 4:** Seasonal distribution of mortality and 90-day events.

Variable	Spring (*n* = 79)	Summer (*n* = 88)	Autumn (*n* = 82)	Winter (*n* = 70)	*p*
Mortality, *n* (%)					
1-Month Mortality	3 (3.8%)	1 (1.1%)	1 (1.2%)	0 (0.0%)	0.316 *
3-Month Mortality	4 (5.1%)	4 (4.5%)	3 (3.7%)	2 (2.9%)	0.926 *
1-Year Mortality ^†^	8/79 (10.1%)	8/88 (9.1%)	9/63 (14.3%)	8/68 (11.8%)	0.773
Events Within the First 90 Days, *n* (%)					
Hospitalization	50 (63.3%)	46 (52.3%)	47 (57.3%)	37 (52.9%)	0.472
ICU Admission	13 (16.5%)	14 (15.9%)	15 (18.3%)	16 (22.9%)	0.684
Sepsis	13 (16.5%)	13 (14.8%)	10 (12.2%)	9 (12.9%)	0.866

* Because expected cell counts were <5 for the 1- and 3-month mortality analyses, the Fisher–Freeman–Halton exact test (the multi-category extension of Fisher’s exact test, calculated by exhaustive enumeration of all 2 × 4 tables with the observed margins) was used instead of the chi-square test for these two rows. ^†^ The 1-year mortality was calculated among 298 patients not excluded for insufficient follow-up; 21 patients who started dialysis between 4 September and 4 December 2025 were excluded from this analysis (autumn *n* = 63, and winter *n* = 68; the other two seasons were unaffected). Three further deaths within one year occurred among these 21 patients (36 deaths in the whole cohort). Follow-up is nominal for survivors (see Methods Section 2.1).

**Table 5:** Effect of season on initiation type, early complications, and 3-month mortality.

Variable	OR	95% CI	*p*
Model 1: Emergent Initiation (*n* = 319; ref: planned; ref season: summer)			
Spring	1.68	0.89–3.15	0.108
Autumn	1.22	0.66–2.25	0.531
Winter	1.82	0.94–3.52	0.076
Age	1.00	0.99–1.02	0.796
Male sex	1.16	0.72–1.86	0.550
Diabetes	0.69	0.42–1.11	0.123
Hypertension	0.98	0.51–1.90	0.955
CVD	0.65	0.39–1.10	0.110
Season, omnibus LRT (3 df)	—	—	0.230
Model 2: Any Early Complication (*n* = 314; ref season: summer)			
**Spring**	**2.33**	**1.23–4.41**	**0.009**
Autumn	1.39	0.75–2.59	0.298
Winter	1.62	0.85–3.10	0.143
Age	0.99	0.97–1.00	0.076
Male sex	0.71	0.44–1.14	0.153
Diabetes	1.05	0.65–1.70	0.832
Hypertension	1.16	0.60–2.24	0.651
CVD	1.48	0.88–2.48	0.140
Season, omnibus LRT (3 df)	—	—	0.069
Model 3: 3-Month Mortality, Firth Penalized Model with Season Only (13 events; *n* = 319; ref season: summer)			
Spring	1.12	0.28–4.48	0.869
Autumn	0.83	0.18–3.50	0.794
Winter	0.69	0.12–3.19	0.634

OR: odds ratio, CI: confidence interval, CVD: coronary artery and/or valvular heart disease. Bold rows indicate statistical significance (*p* < 0.05). Omnibus: likelihood-ratio test of the three season indicators (3 df). Models 1 and 2 are complete-case analyses (Model 2 excludes 5 patients with unknown complication status). Model 3 is a Firth penalized logistic regression with season as the only covariate (profile-likelihood CIs), used because 13 events do not support a multivariable model.

**Table 6:** Seasonal distribution of general demographic and laboratory characteristics.

Variable	Spring (*n* = 79)	Summer (*n* = 88)	Autumn (*n* = 82)	Winter (*n* = 70)	*p*
Demographic Characteristics					
Age, median (Q1–Q3)	59 (49–68)	65 (52–69)	61 (55–69)	66 (53–72)	0.300
Male sex, *n* (%)	53 (67.1%)	50 (56.8%)	45 (54.9%)	51 (72.9%)	0.067
Comorbidities, *n* (%)					
Diabetes	43 (54.4%)	48 (54.5%)	36 (43.9%)	36 (51.4%)	0.480
Hypertension	68 (86.1%)	76 (86.4%)	67 (81.7%)	55 (78.6%)	0.510
Congestive heart failure	22 (27.8%)	27 (30.7%)	34 (41.5%)	21 (30.0%)	0.256
Laboratory Findings, median (Q1–Q3)					
Creatinine (mg/dL)	7.6 (6.1–9.2)	7.1 (6.0–9.1)	7.2 (6.3–8.9)	7.3 (6.0–9.4)	0.976
Urea (mg/dL)	191 (145–238)	176 (142–219)	179 (141–218)	197 (153–241)	0.235
eGFR (mL/min)	7.0 (5.0–9.0)	6.0 (5.0–8.2)	6.5 (5.0–8.0)	7.0 (5.0–8.8)	0.832
Potassium (mEq/L)	4.8 (4.2–5.4)	4.8 (4.3–5.4)	5.0 (4.4–5.7)	4.9 (4.3–5.7)	0.570
Sodium (mEq/L)	136 (133–139)	137 (135–139)	138 (135–140)	137 (135–140)	**0.026**
Hemoglobin (g/dL)	9.2 (8.3–10.0)	8.9 (8.1–9.8)	8.9 (7.7–10.0)	8.9 (8.0–10.1)	0.690
Albumin (g/dL)	3.3 (2.9–3.6)	3.4 (3.0–3.8)	3.4 (2.9–3.8)	3.3 (2.9–3.5)	0.334
pH	7.3 (7.2–7.3)	7.3 (7.2–7.3)	7.3 (7.2–7.3)	7.3 (7.2–7.3)	0.339
HCO3 (mEq/L)	18.0 (14.2–21.0)	18.0 (14.4–22.0)	16.8 (14.0–20.4)	16.9 (13.1–20.1)	0.460
Blood Pressure, median (Q1–Q3)					
Systolic BP (mmHg)	150 (131–166)	142 (130–160)	145 (130–170)	140 (130–159)	0.513
Diastolic BP (mmHg)	80 (73–95)	80 (70–90)	85 (75–98)	78 (70–90)	0.060

Continuous variables are presented as the median (Q1–Q3); *p* values calculated using the Kruskal–Wallis test. Bold value indicates statistical significance (*p* < 0.05).

**Table 7:** Spring versus summer catheter dysfunction: sensitivity analyses.

Analysis	Spring	Summer	OR	95% CI	*p*
Unadjusted comparisons (Fisher’s exact test)					
All patients with known outcome	23/77 (29.9%)	14/87 (16.1%)	2.22	—	0.041
Catheter-exposed patients (primary)	22/70 (31.4%)	13/79 (16.5%)	2.33	—	0.035
Alternative denominator (+2 AVF-start patients with bridging-catheter dysfunction)	23/71 (32.4%)	14/80 (17.5%)	2.26	—	0.038
Excluding starts within 30 days before a season boundary	15/46 (32.6%)	8/57 (14.0%)	2.96	—	0.032
Season assigned at midpoint of 30-day window	20/70 (28.6%)	13/77 (16.9%)	1.97	—	0.114
Excluding initiations Feb–Dec 2023	14/41 (34.1%)	12/61 (19.7%)	2.12	—	0.111
Adjusted for age, sex, diabetes, hypertension, and CVD (logistic regression, catheter-exposed)					
Primary adjusted model (*n* = 278)	—	—	2.30	1.04–5.07	0.039
+Calendar year (*n* = 278)	—	—	3.25	1.35–7.85	0.009
Excluding Feb–Dec 2023 (*n* = 203)	—	—	2.29	0.91–5.73	0.077
By calendar year (catheter-exposed; Fisher’s exact test)					
2022	6/12 (50.0%)	10/29 (34.5%)	1.90	—	0.485
2023	8/29 (27.6%)	1/18 (5.6%)	6.48	—	0.124
2024	6/18 (33.3%)	2/13 (15.4%)	2.75	—	0.412
2025	2/11 (18.2%)	0/19 (0.0%)	not estimable	—	0.126

OR: odds ratio (sample odds ratio for Fisher’s exact tests, for which no CI is shown). Unless stated otherwise, analyses are restricted to catheter-exposed patients with known complication status. Adjusted models include age, sex, diabetes, hypertension, and cardiovascular disease; “+Calendar year” adds the calendar year of initiation as a categorical term. “Alternative denominator” additionally includes the 2 AVF-start patients (spring 1 and summer 1) whose catheter dysfunction occurred in a bridging catheter placed within 30 days.

## Data Availability

The patient-level data are not publicly available due to patient privacy and institutional confidentiality restrictions but are available on request from the corresponding author. The data dictionary (Supplementary Table S3) and the complete analysis code (Supplementary Code File), which contain no patient data, are provided as Supplementary Materials.

## Supplementary Materials

The following supporting information can be downloaded at: https://www.mdpi.com/article/10.3390/jcm15208139/s1, Figure S1: Flow of patients and analytic samples; Table S1: Reproduction of the originally reported models and coding corrections; Table S2: Season effects: omnibus tests, pairwise contrasts, and alternative models; Table S3: Data dictionary (cleaned patient-level dataset); Supplementary Code File: analysis_round3.py (contains no patient data).
