# Supplementary material for: Seasonal Initiation of Hemodialysis: A Single-Center Experience
Source: J Clin Med. 2026 Oct 20;15(20):8139. doi: 10.3390/jcm15208139 (PMC13705731; doi:10.3390/jcm15208139)
Supplement: Supplementary file 1 [file jcm-15-08139-s001.zip › Supplementary_Materials_jcm-4582644.pdf]

Supplementary Materials

Seasonal Initiation of Hemodialysis: A Single-Center Experience (manuscript jcm-4582644). Contents: Figure S1; Supplementary Table S1 (reproduction of the originally reported models and coding corrections); Supplementary Table S2 (omnibus tests, pairwise contrasts, alternative reference seasons, calendar-year-adjusted and sensitivity models); Supplementary Table S3 (data dictionary); Supplementary Code File (analysis_round3.py; contains no patient data).

Figure S1. Flow of patients and analytic samples

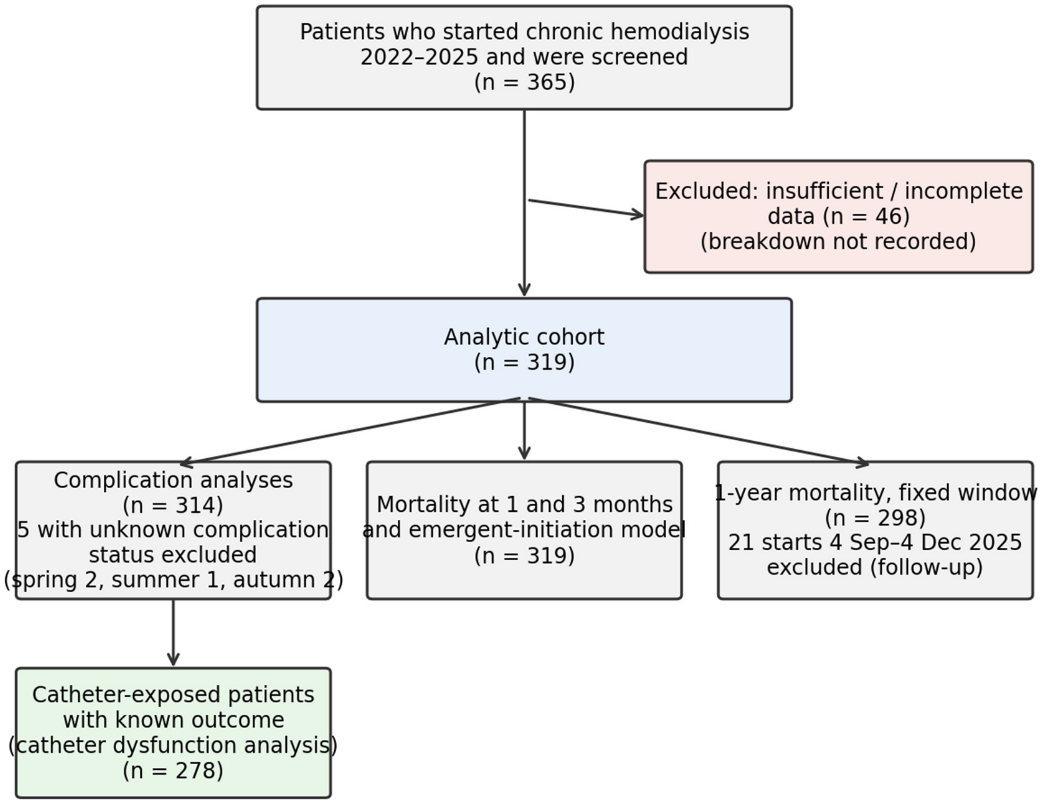

The screening records documented all 46 exclusions as incomplete data; a breakdown by variable and the criteria applied before the pool of 365 was assembled were not recorded.

Supplementary Table S1. Reproduction of the originally reported models and coding corrections

A. Coding corrections identified during the re-audit of the dataset

Item	Coding used in the originally reported models	Corrected coding	Consequence

Cardiovascular disease covariate (CAD and/or valvular disease; one comorbidity field)	Coded 1 only when the field contained exactly "1" or "2"; the 23 patients coded "1,2" were treated as having no disease (148 patients with the covariate)	Any code present: 171/319 (53.6%)	Model 1: CVD OR 0.58 (p=0.033) → 0.65 (0.39–1.10; p=0.110). Model 2: CVD OR 1.04 → 1.48 (0.88–2.48)
Complication outcome in patients with blank (n=4) or "unknown" (n=1) complication field	Blanks counted as no complication; "unknown" counted as a complication (n=319, 164 events)	The 5 patients excluded from complication analyses (n=314, 161 events)	Model 2: spring OR 2.19 (p=0.015) → 2.33 (1.23–4.41; p=0.009); Table 3 autumn 42 → 40 and winter 37 → 36 events (the last decrement of each from the AVF-code correction below); any-complication omnibus p 0.116 → 0.090
Catheter dysfunction code in patients who started with an AVF	Three AVF-start patients (one each in spring, autumn, winter; no access change recorded) carried a catheter dysfunction code	Code removed after review by the authors (no dysfunction occurred); two other AVF-start patients with a bridging catheter retain the code	Any-complication counts: autumn 41 → 40, winter 37 → 36 events; unrestricted catheter dysfunction: spring 24 → 23, autumn 16 → 15, winter 15 → 14; catheter-exposed analyses unchanged
Catheter dysfunction denominators	Restricted analysis counted the 3 unknown outcomes as no dysfunction (22/72 vs 13/80; p=0.053)	Patients with unknown outcome excluded (22/70 vs 13/79; p=0.035)	See Table 7 of the manuscript

B. Models 1 and 2: originally reported estimates (reproduced exactly from the cleaned dataset with the original coding) and corrected estimates; OR (95% CI), p

**Term**	**Originally reported (reproduced)**	**Corrected**	**Note**
Model 1: emergent initiation			
Spring	1.68 (0.90–3.17); 0.106	1.68 (0.89–3.15); 0.108	
Autumn	1.19 (0.64–2.21); 0.576	1.22 (0.66–2.25); 0.531	
Winter	1.81 (0.93–3.51); 0.080	1.82 (0.94–3.52); 0.076	
Age	1.00 (0.99–1.02); 0.736	1.00 (0.99–1.02); 0.796	
Male sex	1.17 (0.72–1.88); 0.529	1.16 (0.72–1.86); 0.550	
Diabetes	0.68 (0.42–1.09); 0.108	0.69 (0.42–1.11); 0.123	
Hypertension	0.99 (0.51–1.91); 0.978	0.98 (0.51–1.90); 0.955	
CVD	0.58 (0.35–0.96); 0.033	0.65 (0.39–1.10); 0.110	
Model 2: any early complication			
Spring	2.19 (1.17–4.11); 0.015	2.33 (1.23–4.41); 0.009	
Autumn	1.48 (0.80–2.74); 0.210	1.39 (0.75–2.59); 0.298	
Winter	1.73 (0.91–3.31); 0.096	1.62 (0.85–3.10); 0.143	
Age	0.99 (0.97–1.00); 0.101	0.99 (0.97–1.00); 0.076	
Male sex	0.68 (0.43–1.09); 0.113	0.71 (0.44–1.14); 0.153	
Diabetes	1.20 (0.75–1.92); 0.445	1.05 (0.65–1.70); 0.832	
Hypertension	1.31 (0.69–2.49); 0.407	1.16 (0.60–2.24); 0.651	
CVD	1.04 (0.64–1.70); 0.865	1.48 (0.88–2.48); 0.140	

Model 3 (3-month mortality; 13 events), originally reported with nine predictors: spring OR 1.15 (0.27–4.94), autumn 0.96 (0.20–4.66), winter 0.63 (0.11–3.75); all reproduced exactly. This model has been replaced by a season-only Firth model (manuscript Table 5).

Supplementary Table S2. Season effects: omnibus tests, pairwise contrasts, and alternative models

A. Model 2 (any early complication, n=314): all six pairwise season contrasts (OR, 95% CI; equivalent to using each season in turn as the reference). Omnibus likelihood-ratio test of season: χ^2=7.10, 3 df, p=0.069.

Contrast	OR (95% CI)	p
Spring vs Summer	2.33 (1.23–4.41)	0.009
Spring vs Autumn	1.68 (0.87–3.21)	0.120
Spring vs Winter	1.44 (0.74–2.80)	0.289
Summer vs Autumn	0.72 (0.39–1.34)	0.298
Summer vs Winter	0.62 (0.32–1.18)	0.143
Autumn vs Winter	0.86 (0.44–1.65)	0.645

Holm-adjusted p values for the three season-versus-summer contrasts: spring 0.028, autumn 0.298, winter 0.287.

B. Model 1 (emergent initiation, n=319): all six pairwise season contrasts. Omnibus LRT: χ^2=4.31, 3 df, p=0.230.

Contrast	OR (95% CI)	p
Spring vs Summer	1.68 (0.89–3.15)	0.108
Spring vs Autumn	1.38 (0.72–2.63)	0.331
Spring vs Winter	0.92 (0.46–1.83)	0.814
Summer vs Autumn	0.82 (0.45–1.52)	0.531
Summer vs Winter	0.55 (0.28–1.06)	0.076
Autumn vs Winter	0.67 (0.34–1.31)	0.242

C. Models additionally adjusted for calendar year (reference 2022)

Term	OR (95% CI)	p
Model 1 (emergent initiation, n=319)		Omnibus season LRT p=0.305
Spring vs summer	1.59 (0.83–3.03)	0.159
Autumn vs summer	1.23 (0.66–2.30)	0.513
Winter vs summer	1.81 (0.92–3.56)	0.085
Year 2023	1.06 (0.56–2.01)	0.863
Year 2024	0.84 (0.43–1.64)	0.612
Year 2025	0.51 (0.26–1.00)	0.049
CVD	0.59 (0.34–1.00)	0.051
Model 2 (any early complication, n=314)		Omnibus season LRT p=0.041
Spring vs summer	2.62 (1.33–5.18)	0.005
Autumn vs summer	1.61 (0.84–3.10)	0.151
Winter vs summer	1.88 (0.95–3.73)	0.071
Year 2023	0.68 (0.36–1.31)	0.253
Year 2024	0.26 (0.13–0.52)	<0.001
Year 2025	0.27 (0.13–0.54)	<0.001
CVD	1.26 (0.73–2.17)	0.410

D. Model 2 sensitivity: patients with unknown complication status counted as having no complication (n=319)

Term	OR (95% CI)	p
Spring vs summer	2.24 (1.19–4.22)	0.012
Autumn vs summer	1.34 (0.72–2.48)	0.351
Winter vs summer	1.66 (0.87–3.18)	0.125

E. Catheter dysfunction among catheter-exposed patients (n=278): adjusted pairwise contrasts (age, sex, diabetes, hypertension, CVD). Omnibus LRT p=0.222.

Contrast	OR (95% CI)	p
Spring vs Summer	2.30 (1.04–5.07)	0.039
Spring vs Autumn	1.53 (0.70–3.32)	0.285
Spring vs Winter	1.45 (0.65–3.21)	0.360
Summer vs Autumn	0.66 (0.29–1.53)	0.335
Summer vs Winter	0.63 (0.27–1.48)	0.291
Autumn vs Winter	0.95 (0.41–2.20)	0.904

F. Firth penalized model for 3-month mortality (13 events), season only; reference summer. Counts (3-month deaths/n): spring 4/79, summer 4/88, autumn 3/82, winter 2/70.

Season	OR (95% CI)	p
Spring	1.12 (0.28–4.48)	0.869
Autumn	0.83 (0.18–3.50)	0.794
Winter	0.69 (0.12–3.19)	0.634

G. Holm correction across the family of 33 seasonal omnibus tests (Tables 3, 4, and 6): smallest unadjusted p=0.026 (serum sodium); smallest Holm-adjusted p=0.858; all other adjusted p values =1.000.

Supplementary Table S3. Data dictionary (cleaned patient-level dataset)

Column	Variable	Coding / remarks
0	Patient name	Direct identifier; not used in any analysis; removed from analysis files
1	Age	Years
2	Sex	1=female, 2=male
3	Date of first hemodialysis session	Calendar date
4	Month of initiation	1–12
5	Season of initiation	1=spring (Mar–May), 2=summer (Jun–Aug), 3=autumn (Sep–Nov), 4=winter (Dec–Feb)
6	Initiation type	1=planned, 2=emergent (clinical classification by treating team)
7	Initial vascular access	1=AVF, 2=tunneled catheter, 3=temporary catheter
8	Change of vascular access within first 30 days	0=no, 1=yes
9	Primary kidney disease	Free text / category
10	Diabetes	0/1
11	Hypertension	0/1
12	Congestive heart failure	0/1
13	Coronary artery and/or valvular heart disease	Codes: 1=coronary artery disease, 2=valvular disease, "1,2"=both; model covariate = any code present
14	Chronic lung disease	0/1
15	Stroke	0/1
16	Peripheral arterial disease	0/1
17	Early nephrology referral	1=yes, 0=no (not analyzed in this study)
18	Creatinine	mg/dL
19	Urea	mg/dL
20	eGFR	mL/min
21	Potassium	mEq/L
22	Sodium	mEq/L
23	Hemoglobin	g/dL

24	Albumin	g/dL
25	pH	
26	HCO3	mEq/L
27	Pro-BNP	Missing for 180 patients; not analyzed
28	Systolic blood pressure	mmHg, single pre-dialysis value
29	Diastolic blood pressure	mmHg, single pre-dialysis value
30	Parathyroid hormone	Not analyzed
31	ESA use	0/1 (not analyzed)
32	ACE-inhibitor/ARB use	0/1 (not analyzed)
33	Pre-dialysis volume status	1=hypervolemic, 2=normovolemic, 3=hypovolemic (not analyzed)
34	Oliguria/anuria	0=none, 1=oliguria, 2=anuria (not analyzed)
35	Pre-dialysis infection	0/1 (not analyzed)
36	Infection type	1=pneumonia, 2=urinary, 3=other (not analyzed)
37	Causative organism (pre-dialysis infection)	Free text (not analyzed)
38	Emergency HD indication present	0/1
39	Volume overload	0/1
40	Metabolic acidosis	0/1
41	Hyperkalemia	0/1
42	Uremic symptom	0=none, 1=anorexia, 2=nausea, 3=pruritus, 4=encephalopathy, 5=bleeding (not analyzed)
43	Length of hospital stay (days)	Not analyzed
44	Dialysis complication(s) within 30 days	0=none, 1=catheter infection, 2=catheter dysfunction, 3=AV fistula thrombosis, 4=intradialytic hypotension, 5=other; several codes may be recorded ("1,2"); blank or "unknown" = unknown status
45	Organism, catheter infection	Free text (not analyzed)
46	Catheter exchange after catheter infection	0/1 (not analyzed)
47	Catheter exchange due to dysfunction	0/1; derived during data cleaning; identical to the catheter dysfunction code
48	Hospitalization within 90 days	0/1
49	ICU admission within 90 days	0/1
50	Cardiac complication within 90 days	0/1 (not analyzed)
51	Sepsis within 90 days	0/1
52	Death within 1 month	0/1
53	Death within 3 months	0/1
54	Death within 1 year	0/1
55	Follow-up (months)	For decedents: month of death; for survivors: nominal 12 (one patient 22); not an observed time to last contact
56	Data-cleaning note	Free text from the cleaning log
57	Date (cleaned)	Calendar date of first session after cleaning
58	Eligible for 1-year mortality analysis	True/False; False for the 21 patients starting 4 Sep–4 Dec 2025
